# Prevalence of biologics monotherapy in a cohort of patients with Rheumatoid Arthritis in daily clinical practice

**DOI:** 10.1186/s12891-016-0959-1

**Published:** 2016-03-01

**Authors:** Erika Catay, Maximiliano Bravo, Javier Rosa, Enrique R. Soriano

**Affiliations:** Sección Reumatologia, Servicio de Clinica Médica, Hospital Italiano de Buenos Aires, and Instituto Universitario del Hospital Italiano de Buenos Aires, Peron 4190, CABA, (1181) Buenos Aires, Argentina; Fundacion P.M. Catoggio para el Progreso de la Reumatologia, Buenos Aires, Argentina

**Keywords:** Rheumatoid Arthritis, Biologic therapy, Monotherapy, Drug survival

## Abstract

**Background:**

Real-life registry data reveal approximately one-third of patients taking biologic agents use them as monotherapy, in spite that combination therapy with Disease Modifying Drugs is more efficacious than monotherapy. The aim of our study was to assess the prevalence of biologics monotherapy in a cohort of patients with RA followed at a single center, and to analyze the reasons for monotherapy, including patients with prescriptions that do not take the medication.

**Methods:**

All patients with Rheumatoid Arthritis, with biologic therapy followed at our Rheumatology Unit were included. Prevalence and reasons for biologics monotherapy was calculated in general, for each biologic course and for each biologic. Prescription data was obtained from the Electronic Medical Record, and drugs acquisition was obtained from the Hospital Administrative database. Drug survival was also calculated and compared between monotherapy and combination therapy.

**Results:**

Seventy nine patients with 115 courses of biologic treatments were included. In 40 (35 %, 95 % CI: 26–44 %) of all biologics courses, biologics were initiated as monotherapy. In 27 courses (23 %, 95 % CI: 16–32 %) biologic monotherapy was prescribed by the treating rheumatologists, and in the other 13 (11 %, 95 % CI: 6–18 %) it was initiated as such by decision of the patient regardless of the physician indication. Reasons for prescription of biologic monotherapy by the treating rheumatologists were adverse events with previous DMARDs in 55.5 %, and was not specified in the remaining courses. Only 25 % of biologics’ courses were monotherapy from the beginning to the end of the biologic therapy. The overall survival on biologics was 45 % (95 % CI: 35–55 %) at 3 years. There were no statistically differences in biologics survival by modality (monotherapy vs combination) (*p* = 0.543), course (*p* = 0.4454), or by biologic drug (*p* = 0.9612).

**Conclusions:**

Almost 1/3 of patients on biologics use them as monotherapy. This is due to physician’s preferences in 60 % of the cases, and to patients not compliance with the indication in around 40 % of the cases. Better communications is needed to assure that physicians and patients agree on the prescribed and used medication.

## Background

The importance of early therapeutic intervention to relieve symptoms, prevent newly evolving joint erosions and joint space narrowing, improve functional abilities and quality of life in patients with active RA is well established [1, 2]. The conventional DMARD MTX is the standard of care for patients with RA, however there is still a substantial number of patients that do not respond to MTX [1, 3]. On the other hand there are patients with RA that are intolerant to MTX, drug interactions preclude the use of MTX with certain medications, and some patients experience toxicity or adverse events.

The efficacy of biologic agents in combination with MTX is well established and that appears to be the most effective regimen currently available for patients with early or established RA who have failed to respond to traditional DMARDs [2]. However, biologic monotherapy is commonly used in clinical practice. Data from biologic registries and US claims databases indicate that approximately 30 % of patients taking biologics use them as monotherapy [4–7]. However, this figure does not capture patients who fill prescriptions but do not take some or all of the medication [6]. Many patients in whom MTX or other traditional DMARDs are prescribed in combination with biologics decide not to take them, very often, without telling their Rheumatologist. In an online survey of 1500 patients, 45 % admitted to being less than forthright with their rheumatologists [8].

The purpose of our study was to assess the prevalence of biologics monotherapy in a cohort of patients with RA followed at a single center, and to analyze the reasons for monotherapy, including patients with prescriptions that do not take the medication.

## Methods

All patients with RA affiliated to the Hospital Italiano de Buenos Aires Health Management Organization (Plan de Salud) treated with biologics were included, in a retrospective observational cohort study.

Patients fulfilled RA 1987 [9] or 2010 [10, 11] classification criteria, and were treated with one of the following biologic agents: TNF inhibitors (TNFi) (infliximab, etanercept, adalimumab, certolizumab-pegol or golimumab), anti-CD20 antibody (rituximab), a cytotoxic T lymphocyte antigen-4 fusion protein (abatacept) or an IL-6 receptor (IL-6R) antagonist (tocilizumab).

All patients with biologic treatment without exceptions, are register in our Unit’s registry of biologics, and in the HMO administrative databases,, as it is a requirement to get the therapy. Treatment was decided by the treating rheumatologists, according to National guidelines. Patients must fail at least one DMARD to receive biologic therapy. The clinical data of all patients included was reviewed and extracted from ours Hospital Electronic Medical records and our register. Demographic data, dates on diagnostic, initial prescription of DMARD therapy (DMARDs included were: Methotrexate, leflunomide, sulfasalazine, and Hydroxychloroquine), initial prescription of biologics, subsequent change of DMARD (on top of the biologic), reasons for the change, and last visit on follow up were collected. The number of DMARDs and biologics actually acquired by each patient were captured from the HMO administrative databases, in order to assess the compliance of each patient with the prescribed medication during the 6–12 months before and after the acquisition of the prescribed biologic drug. All medications acquired by patients affiliated to our HMO are registered in the HMO administrative database, without exception. Patients could only acquire DMARDs and biologics in Argentina with a prescription. Hospital Italiano HMO has their own pharmacies, where medications prescribed are sold to the patients at a lower cost, and all transactions are registered in the HMO administrative data base. Patients could also buy their medications in other pharmacies adhered to the Hospital Italiano HMO, where they buy them with an important discount of 40 to 50 % of medication price, and this transaction is also registered in the Hospital Italiano HMO administrative database. Medications that are used chronically are prescribed by the treating physicians in the electronic medical records, and usually acquired by the patients at the HMO’s pharmacies. The only circumstance in which a medication acquired by a patient would not be registered is if the patients buys it in a private pharmacy paying the medication full price. HMO’s private studies and surveys (unpublished information) have shown that 70 % of chronic medications are acquired in the HMO pharmacies, and that 98 % of all medications are acquired within the HMO system.

The study was conducted according to the Declaration of Helsinki and local regulations. Ethical approval for the study was obtained from the Hospital local Ethics Committee (comité de ética y protocolos de investigación (CEPI). As data was obtained from Electronic Medical Records, and administrative data, the Ethics committee considered that an informed consent was not needed. All patients at entrance to the Hospital Italiano HMO signed an informed consent authorizing the use of their data for clinical research.

### Statistical analysis

The prevalence of monotherapy was calculated with its 95 % confidence intervals, in general and for each biologic drug and for each treatment course. Analysis was done at course treatment level, (a patient with more than one course of biologic treatment was included more than once in this analysis). A biologic treatment course was defined as the use of the biologic drug since first prescribed until the drug was definitively stopped (if the drug was temporarily discontinued it was considered the same treatment course). First course was the first biologic prescribed, second course was the second biologic prescribed, etc.

Biologics monotherapy at initiation of the biologic treatment was defined in two different ways:

1. Prescribed monotherapy: course of treatment in which the treating Rheumatologist prescribed the biologic without concomitant DMARD; data obtained from the Electronic Medical Record.
2. Actual monotherapy: patients taking biologic as monotherapy in that course of treatment, independently of the treating Rheumatologist prescription, as obtained by data on DMARDs acquisition from HMO administrative database;
3. Prevalence of monotherapy during the complete biologic treatment was also calculated, as some patients started biologics monotherapy but then a DMARD was added.

Reasons for monotherapy was listed and categorized in toxicity/intolerance to DMARD, reluctance of the patients to receive concomitant DMARDs and Physician decision.

Kaplan–Meier survival curves on different biologics and for patients in monotherapy and in combination, using Breslow (generalized Wilcoxon) test for comparison among different groups were constructed. In addition, the median (IQR) durations of intake of various biologics and between monotherapy and combination therapy was compared using Kruskal Wallis test. A *p*-value < 0.05 was considered to be significant for all statistical comparisons

## Results

Seventy-nine patients with 115 courses of biologic treatments were included. Patients’ characteristics are shown in Table 1. All patients have received at least one DMARD before biologics. Most patients (54 %) were receiving combination therapy (of which the most frequent combination was MTX plus LFN) at the time of starting biologics, 27 % were receiving MTX monotherapy and 11 % were receiving LFN monotherapy.

**Table 1:** Baseline patient characteristics

Patient characteristics	Total population
Sex, n female (%)	71 (92)
Mean age, years (SD)	62.7 (14)
Mean disease duration, years (SD)	14 (9.1)
Mean DAS28 (SD)	5.47 (0.84)
Mean HAQ (SD)	1.4 (1.2)
Rheumatoid factor positive, %	92
Anti CCP antibodies positive, %	75,3
Median follow up (IQ range), Yrs	1.2 (0.5–3)
Median number of DMARDs before biologics (IQ range)	2 (1–3)

Taking into account all courses of biologic treatments, in 40 (35 %, 95 % CI: 26–44 %) biologics were initiated as monotherapy. In 27 courses (23 %, 95 % CI: 16–32 %) biologic monotherapy was prescribed by the treating rheumatologists, and in the other 13 (11 %, 95 % CI: 6–18 %) it was initiated as such by decision of the patient regardless of the physician indication (Tables 2 and 3). Reasons for prescription of biologic monotherapy by the treating rheumatologists were adverse events with previous DMARDs in 15/27 courses (55.5 %), and was not specified in the remaining coues. There were some differences among the different biologics related to the percentage of monotherapy that was due to rheumatologists prescription compared to patient’s decision. TNFi, rituximab, abatacept and tocilizumab were prescribed as monotherapy by the treating rheumatologist in 77 %, 50 %, 25 % and 60 % of the courses of monotherapy respectively. Some patients that initiated the biologic course as monotherapy added traditional DMARDs during treatment. Only 29/115 (25 %) courses of biologic therapy were as monotherapy from the beginning to the end of the biologic therapy. Among the 40 monotherapy courses, 29 (72.5 %) continued as monotherapy for the complete course, while in the others a traditional DMARD was added (Table 4). On the other hand some patients initiated MTX with biologic therapy in combination, and subsequently discontinue it due to adverse events, or other causes, so we also looked at the prevalence of monotherapy with biologics at the end of the biologic course (or last follow up for those patients not stopping/switching biologics) that was 33 % (95 % CI: 24–42) (Table 5).

**Table 2:** Percentage of patients on monotherapy according to treatment course and drug

	1^st^ Course	2^nd^ Course	3^rd^ Course	4^th^ Course	Total
Number of patients	79	25	9	2	115
% Patients on biologic monotherapy at initiation of biologics (95 % CI)	24 (15–35)	60 (39–79)	44 (14–79)	100	35 (26–44)
% Patients on biologic monotherapy at initiation of biologics due to Medical prescription (95 % CI)	15 (8–25)	48 (28–69)	33 (7–70)	0 (0)	23 (16–32)
% Patients biologic monotherapy at initiation of biologics, due to patient’s decision (95 % CI)	9 (3.6–17)	12 (2.5–31)	11 (0.2–48)	100	11 (6–18)

**Table 3:** Percentage of patients on monotherapy according to the biologic treatment

	1st Course	2nd Course	3rd Course	4th Course	Total
N Patients on adalimumab monotherapy/patients on adalimumab (%)	5/27 (18)	2/3(67)	-	-	7/30 (23)
N patients on etanercept monotherapy/patients on etanercept (%)	12/39 (31)	4/6 (67)	1/1 (100)	-	17/46 (37)
N patients on Infliximab monotherapy/patients on Infliximab (%)	0/7(0)	1/1 (100)	0/0	0/0	1/8 (12)
N patients on Abatacept monotherapy/patients on Abatacept (%)	1/1 (100)	6/10 (60)	0/3 (0)	1/1 (100)	8/15 (53)
N patients on Rituximab monotherapy/patients on Rituximab (%)	1/5 (20)	0/2 (0)	0/1 (0)	1/1 (100)	2/9 (22)
N patients on Tocilizumab monotherapy/patients on Tocilizumab (%)	0/0 (0)	2/2 (100)	3/4 (75)	0/0 (0)	5/6 (83)

**Table 4:** Percentage of patients on monotherapy during the complete biologic course, according to biologic treatment course

	1^st^ Course	2^nd^ Course	3^rd^ Course	4^th^ Course	Total
Number of patients	79	25	9	2	115
% Patients on biologic monotherapy during the complete biologic course (95 % CI)	15 (8–25)	48 (28–69)	44 (14–79)	50 (13–99)	25 (18–34)
% Patients on biologic monotherapy during the complete biologic course due to Medical prescription (95 % CI)	10 (4–19)	40 (21–61)	33 (7–70)	0	18 (12–26)
% Patients biologic monotherapy during the complete biologic course due to patient’s decision (95 % CI)	5 (1–12)	8 (1–26)	11 (0.2–48)	50 (13–99)	7 (3–13)

**Table 5:** Percentage of patients on monotherapy at the end of biologic course, according to the biologic treatment course

	1^st^ Course	2^nd^ Course	3^rd^ Course	4^th^ Course	Total
Number of patients	79	25	9	2	115
% Patients on biologic monotherapy at the end of the course (95 % CI)	25 (16–36)	52 (31–72)	44 (14–79)	50 (13–99)	33 (24–42)
% Patients on biologic monotherapy at the end of the course due to Medical prescription (95 % CI)	20 (12–31)	40 (21–61)	33 (7–70)	50 (13–99)	25 (17–34)
% Patients biologic monotherapy at the end of the course due to patient’s decision (95 % CI)	5 (1–12)	12 (2.5–31)	11 (0.3–48)	0	8 (4–14)

**Fig. 1:**
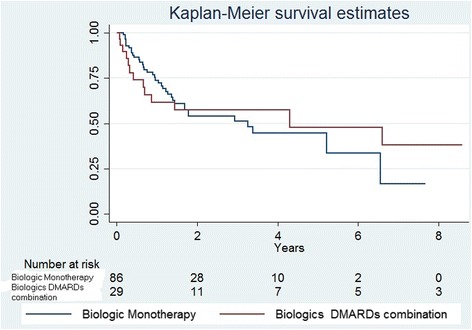
Biologics survival by Biologic therapy modality: Monotherapy throughout all the course of biologic therapy vs biologic therapy combined with DMARDs

Median follow up of this cohort was 1.2 years (IQR: 0.5–3). The overall survival on biologics was 45 % (95 % CI: 35–55 %) at 3 years and 39 % (95 % CI: 28–50 %) at 4 years. The survival by biologic are shown in Table 6. There were no statistically differences in biologics survival by course (*p* = 0.4454), or by biologic drug (*p* = 0.9612). There were no statistical differences on drug survival at 3 years between patients on monotherapy throughout the complete course and those with combined therapy (58 %; 95 % CI: 37–74, vs 52 %; 95 % CI: 39–63, respectively) (Fig. 1).

**Table 6:** Drug survival by biologic drug

Biologic drug	Interval	% biologics’ survival (95 % CI)
Etanercept	3 years	59 (41–73)
Infliximab	3 years	20 (2–52)
Adalimumab	3 years	54 (33–70)
Rituximab	3 years	45 (10–75)
Abatacept	3 years	63 (33–83)
Tocilizumab	2 years	75 (13–96)

## Discussion

Concurrent use of MTX and a biologic is generally the standard-of-care in patients with RA who continue with disease activity despite MTX. Many patients, however, do not take MTX concomitantly as prescribed. This figure however is not clear, as few studies have investigated that prevalence.

We found that 35 % of patients started their biologic therapy as monotherapy. Among them 11 % used monotherapy because they did not take traditional DMARDs concomitantly as prescribed, 23 % of patients initiating a biologic therapy did so as monotherapy due to the Rheumatologist prescription.

A recent study on 6744 patient records in private and public practice in Canada showed that among patients on their first biologics, 45 % did not purchase a DMARD. By contrast, physicians reported that they prescribed a DMARD with a biologic in 80–90 % of patients [6, 12]. Another study on 1652 patient records also in Canada showed biologic monotherapy prescribing rate of 12 %, but 29 % did not acquired their DMARD within 6 months after starting biologic therapy [6, 13]. In the US, Yazici et al. found that 30 % of patients initiating biologics did that as monotherapy in a study involving a large database of patients with newly diagnosed RA [7]. Only 42 % of patients had filled a traditional DMARD prescription during the 6 months prior to initiating the TNFi. In summary, these studies showed that a high percentage of patients did not purchase the prescribed DMARD when they are taking biologics.

In a US study of 7074 biologic naïve patients, found that up to 31 % of patients receiving an anti TNF agent in the real word received it as monotherapy [14].

In CORRONA registry on 9905 patients, 25 % received biologic monotherapy. Among patients that were previously biologic naïve, 19 % initiated a biologic as monotherapy, whereas monotherapy initiation rates for patients who received one prior biologic was 29 %; two prior biologics 26 %; and three or more prior biologics 31 %. Prior biologic experience and individual physician’s prescribing patterns were associated with increased likelihood of initiating a biologic as monotherapy [15].

Interestingly in our study physicians, more often prescribed Biologics as monotherapy in the 2^nd^ and 3^rd^ biologic course than in the 1^st^ (48, 33 and 15 % respectively). However, the number of patients not purchasing the traditional DMARD was similar among all treatment courses (9, 12 and 11 % for the 1^st^, 2^nd^, and 3^rd^ courses respectively). Survival on drug therapy was shorter than in clinical trials, but similar to that found in some other registries. We did not find differences on drug survival among patients on biologic monotherapy and combination therapy, but our study was underpowered to detect such differences. More than 2000 patients would be required to show significant statistical differences with the survival rate we found. Survival on drug therapy in some way is a surrogate of efficacy and absence of toxicity. In that sense as we do not have data on efficacy, survival on the drugs seemed to be showing similar efficacy for patients on combination and on monotherapy.

Our study has some strength. We combined clinical records with full administrative data, providing information on actual use of concomitant DMARDs. We also included data from different treatment courses, showing that with each biologic course patients are more willing to receive the biologic treatment as monotherapy. On the other side our study has some weakness, mainly the small number of patients and short time of follow up, and the lack of data on efficacy. Another weakness is that this is a single center study, so results might not be extrapolated to other settings.

## Conclusions

In summary we found that almost 1/3 of patients on biologics use them as monotherapy. This is due to physician’s preferences in 60 % of the cases, and to patients not compliance with the indication in around 40 % of the cases. Better communications is needed to assure that physicians and patients agree on the prescribed and used medication.

## Abbreviations

Anti CCP: anti-cyclic citrullinated peptide autoantibodies
CI: confidence interval
DAS28: disease activity score 28 joints using erythrosedimentation rate
DMARD: disease modifying antirheumatic drug
EMR: electronic medical record
HAQ: health assessment questionnaire
HMO: health management organization
IL-6R: IL-6 receptor
IQ: interquartile range
MTX: methotrexate
TNFi: tumor necrosis factor inhibitors
US: United States
